# A Case of Long-Continued Priapism Accompanying Leukæmia

**Published:** 1879-09

**Authors:** 


A Case of Long-continued Priapism Accompanying Leukæmia.—Dr. Salzer, of Worms, reports the case of a man, 46 years of age, who suffered for seven weeks from persistent priapism He had previously suffered from intermittent fever, but was at this time in apparent good health. One morning he was awakened by an intensely painful erection of the penis, that proved utterly rebellious to treatment. Leeches, warm fomentations, chloral hydrate, and even chloroform narcosis were tried in turn, but all without success. The urine was passed with difficulty, usually in short jets, and most readily in the knee-elbow position. Physical examination revealed only marked enlargement of the spleen. Finally, after the penis had been kept for three weeks constantly enveloped in strongly camphorated narcotic poultices, opium and camphor being administered internally at the same time, the priapism gradually disappeared, having persisted fully seven weeks. During tl e week preceding this attack, the patient had had two attacks of priapism, one of which lasted only a few hours, and the other twenty-four hours. After the appearance of the priapism the patient rapidly lost strength and acquired a cachectic appearance, and the spleen progressively increased in size. Two months after the priapism disappeared there was complete loss of sexual power, and the patient died about eight months afterward. The blood was not examined microscopically, and an autopsy was not permitted.

Dr. Salzer collates from medical literature eight cases of priapism occurring in connection with leukæmia. Various theories have been brought forward to account for the priapism in these cases. Kremme ascribed it to extravasation of blood into the corpora cavernosa, and Longuet to impeded circulation in the smaller vessels and the formation of thrombi, resulting from the altered condition of the blood, while Neidhart thought that irritation of the nerves might possibly be the exciting cause. Dr. Salzer thinks that the rapid disappearance of the priapism in the two first attacks in the above case argues against the occurrence of an extravasation of blood. He believes that both the tempoary and the persistent attacks of priapism were due to irritation of the nervi errigentes. It is well known that priapism may be produced both by peripheral and by central irritation of these nerves. As examples of the former, he adduces the erections accompanying inflammation of the urethra or of the neck of the bladder, swelling of the prostate, etc.; and as examples of the latter, the erections of insane persons, or that follow injuries of spinal cord. The priapism of leukæmia, he claims, differs, from these varieties chiefly in its longer duration, and hence for its development some special cause must be sought. This may possibly be found either in the presence of anatomical changes in the nervi errigentes, or in pressure on them, ex. gr., by swolen lumber glands.—*Med.-Chair Rundschau.*

